# Evidence and consensus based guideline for the management of delirium, analgesia, and sedation in intensive care medicine. Revision 2015 (DAS-Guideline 2015) – short version

**DOI:** 10.3205/000223

**Published:** 2015-11-12

**Authors:** DAS-Taskforce 2015, Ralf Baron, Andreas Binder, Rolf Biniek, Stephan Braune, Hartmut Buerkle, Peter Dall, Sueha Demirakca, Rahel Eckardt, Verena Eggers, Ingolf Eichler, Ingo Fietze, Stephan Freys, Andreas Fründ, Lars Garten, Bernhard Gohrbandt, Irene Harth, Wolfgang Hartl, Hans-Jürgen Heppner, Johannes Horter, Ralf Huth, Uwe Janssens, Christine Jungk, Kristin Maria Kaeuper, Paul Kessler, Stefan Kleinschmidt, Matthias Kochanek, Matthias Kumpf, Andreas Meiser, Anika Mueller, Maritta Orth, Christian Putensen, Bernd Roth, Michael Schaefer, Rainhild Schaefers, Peter Schellongowski, Monika Schindler, Reinhard Schmitt, Jens Scholz, Stefan Schroeder, Gerhard Schwarzmann, Claudia Spies, Robert Stingele, Peter Tonner, Uwe Trieschmann, Michael Tryba, Frank Wappler, Christian Waydhas, Bjoern Weiss, Guido Weisshaar

**Affiliations:** 1 German Society of Neurology (DGN); 2 German Society of Internal Medicine Intensive Care (DGIIN); 3 German Society of Anaesthesiology and Intensive Care Medicine (DGAI); 4 German Society of Gynecology & Obstetrics (DGGG); 5 German Society of Neonatology and Pediatric Intensive Care (GNPI); 6 German Society of Geriatrics (DGG); 7 German Society for Thoracic and Cardiovascular Surgery (DGTHG); 8 German Sleep Society (DGSM); 9 German Society of Surgery (DGCH); 10 German Association for Physiotherapy (ZVK); 11 German Society of Neurosurgery (DGNC); 12 German Society of Midwifery Science (DGHWi); 13 German Society of Haematology and Oncology (DGHO); 14 German Pain Society (DGSS); 15 German Society for Specialised Nursing and Allied Health Professions (DGF); 16 German Association for Psychiatry, Psychotherapy and Psychosomatics (DGPPN); 17 German Interdisciplinary Association for Intensive Care and Emergency Medicine (DIVI)

**Keywords:** guideline, evidence, analgesia, sedation, delirium, anxiety, stress, sleep, monitoring, treatment, intensive care, critical care, Germany

## Abstract

In 2010, under the guidance of the DGAI (German Society of Anaesthesiology and Intensive Care Medicine) and DIVI (German Interdisciplinary Association for Intensive Care and Emergency Medicine), twelve German medical societies published the “Evidence- and Consensus-based Guidelines on the Management of Analgesia, Sedation and Delirium in Intensive Care”. Since then, several new studies and publications have considerably increased the body of evidence, including the new recommendations from the American College of Critical Care Medicine (ACCM) in conjunction with Society of Critical Care Medicine (SCCM) and American Society of Health-System Pharmacists (ASHP) from 2013. For this update, a major restructuring and extension of the guidelines were needed in order to cover new aspects of treatment, such as sleep and anxiety management. The literature was systematically searched and evaluated using the criteria of the Oxford Center of Evidence Based Medicine. The body of evidence used to formulate these recommendations was reviewed and approved by representatives of 17 national societies. Three grades of recommendation were used as follows: Grade “A” (strong recommendation), Grade “B” (recommendation) and Grade “0” (open recommendation). The result is a comprehensive, interdisciplinary, evidence and consensus-based set of level 3 guidelines. This publication was designed for all ICU professionals, and takes into account all critically ill patient populations. It represents a guide to symptom-oriented prevention, diagnosis, and treatment of delirium, anxiety, stress, and protocol-based analgesia, sedation, and sleep-management in intensive care medicine.

## Zusammenfassung

Die vorherige Version der S3-Leitlinie „Analgesie, Sedierung und Delirmanagement in der Intensivmedizin“ wurde 2010 unter der Federführung der Deutschen Gesellschaft für Anästhesiologie und Intensivmedizin (DGAI) und der Deutschen Interdisziplinären Vereinigung für Intensiv- und Notfallmedizin (DIVI) publiziert. Neue Evidenz aus Studien ebenso wie neue Leitlinien, u.a. die 2013 erschienene Leitlinie der U.S.-amerikanischen Society of Critical Care Medicine (SCCM), des American College of Critical Care Medicine (ACCM) und der American Society of Health-System Pharmacists (ASHP), gaben nicht nur Anlass zu einem Update der deutschen Empfehlungen von 2010. Für die Fortschreibung der S3-Leitlinie wurden eine Neuformulierung von klinisch relevanten Schlüsselfragen und die signifikante Erweiterung der Leitlinie um neue Facetten der Behandlung, wie zum Beispiel das Schlafmanagement, notwendig. Dazu wurde die systematisch gesuchte Literatur nach Kriterien des Oxford Centre of Evidence Based Medicine bewertet. Der enorme Evidenzkörper bildete die Grundlage für die Empfehlungen, die von Mandatsträgern aus 17 Fachgesellschaften konsentiert wurden. In den Empfehlungen wurden die Grade „A“ (starke Empfehlung), „B“ (Empfehlung) und „0“ (offene Empfehlung) gewählt. Als Ergebnis dieses Prozesses liegt nun die weltweit umfassendste, interdisziplinär erarbeitete evidenz- und konsensbasierte Stufe 3 Leitlinie vor. Die Leitlinie richtet sich an alle auf der Intensivstation tätigen Berufsgruppen, die Empfehlungen berücksichtigen alle intensivmedizinisch-behandelten Patientengruppen. Sie stellt einen Leitfaden zur symptomorientierten Prävention, Diagnostik und Therapie von Delir, Angst, Stress und der protokollbasierten Analgesie, Sedierung und dem Schlafmanagement in der Intensivmedizin für Erwachsene und Kinder dar.

## Introduction

In 2010, twelve German medical societies published the *Evidence- and Consensus-based Guidelines on the Management of Analgesia, Sedation and Delirium*. Since this publication, the body of evidence in the field has increased considerably. Several new studies and publications, including the new recommendations from the American College of Critical Care Medicine (ACCM) in conjunction with Society of Critical Care Medicine (SCCM) and American Society of Health-System Pharmacists (ASHP) in 2013, make a substantial actualization of the German guidelines necessary. This update not only accounts for new evidence, but also contains a major restructuring and extension of the guidelines to cover new aspects of treatment, such as sleep and anxiety management.

This new version aims to provide practical guidance for the symptom-based prevention, diagnostics and therapy of delirium, anxiety, and agitation, as well as for the protocol-based analgesia, sedation, and sleep management during critical illness. Under the guidance of the German Society of Anaesthesiology and Intensive Care Medicine (DGAI) and German Interdisciplinary Association for Intensive Care and Emergency Medicine (DIVI), this is one of the most comprehensive guidelines worldwide, being developed and approved by 17 national societies.

These guidelines were developed for all professions working in the intensive care unit (ICU), and its recommendations encompass critically ill patients of all age groups and severity of illness, regardless of comorbidities.

Delirium and coma are the most common manifestations of acute brain dysfunction during critical illness. Pain, stress, and a disruption of the sleep-wake-cycle are typical symptoms observed during ICU treatment, all of which can lead to delirium and impair outcome. An early management of these symptoms improves recovery and long-term outcome, while reducing post-intensive-care-unit-syndrome (PICS) and mortality. The basic principle follows an “early goal directed therapy” (EGDT) with immediate and evidence-based targets for treatment, the assessment of the clinical situation with validated instruments, and the goal-directed pharmacological therapy. These measures are to be accompanied by non-pharmacological interventions aimed at prevention and treatment.

**The critically ill patient should be awake and alert, without pain, anxiety, or delirium. Ultimately, this allows the patient to actively participate in their treatment and recovery.**

The term “sedation” was left in the title intentionally: the indication and conduction of sedation require special attention in order not to harm the patient through oversedation, as this has been proven to negatively affect patient outcome. In the interest of the patients, the use of sedatives and analgesics must therefore be very carefully considered. The patient must be allowed to be as alert and oriented as possible, so that they may partake in the therapy and convalescence process, as evidence shows is feasible, practical, and safe.

## Methods

This guideline has the highest development credentials, S3, indicating that it is both evidence- and consensus based. The guideline task-force consisted of 49 voting members nominated by 17 participating national societies. These members formed work groups that identified main clinical issues and framed key-questions to be addressed. Should previous versions of the guideline not /no longer provide satisfactory resolutions to the selected topics, search strategies were developed to address the subject. The resulting recommendations were then collected in an early version and further discussed within the groups. The members interacted with each other via consensus-conferences, email, fax, or mail. Communication was managed by a coordinating group consisting of 4 members, as well as the chairing societies. A systematic literature search was performed by a special team under the supervision of an epidemiologist. Finally, the evaluation process was done by the individual working groups, which also set the level-of-evidence (LoE). All searches were performed between April and May of 2014. When necessary, new evidence was integrated manually. The LoE was determined, as in the previous version of the guideline, using the Oxford System [1]. The overview tables allow to identification the evidence for each individual manuscript. Literature was accessible for all task-force members. The specific search strategies, searched terms, inclusion and exclusion criteria, as well as exact time frames, are all detailed in the methodological report.

Sources were electronic databases (Medline^®^), guideline networks, and (manually) abstracts and congress-publications. In order to identify “grey literature”, all task-force members were asked to search for publications outside Medline and Embase and include relevant entries to the literature-data-base. Systematic guideline searches were conducted within the AWMF-registry (http://www.awmf.org/leitlinien.html) and the Guidelines International Network (G-I-N) (http://www.g-i-n.net). In line with the AWMF-guideline developer manual, a consensus-based decision was used to assess whether recommendations from other guidelines could be adapted. Cultural adaptions were adopted in accordance with recommendations from the *ADAPTE-Collaboration* [2].

Voting only took place following a full disclosure of potential conflicts of interest by the task-force members. The conflict of interest forms were stored centrally, and all task-force members declaring a conflict of interest were required to abstain from voting in the corresponding issues. This was consented in the task-force before the voting process. The voting-process itself was conducted during consensus-conferences, under the supervision of an independent observer from the AWMF. Alternatively, members could vote using an online DELPHI, as published and recommended in the AWMF-guideline developer manual. The used grades of recommendation (GoR) were A = strong recommendation (we recommend/one shall), B = recommendation (we suggest/one should), and O = open recommendation (one might consider) for or against any specific intervention. Significant deviations between LoE and GoR were generally possible if a member of the group requested upgrading or downgrading of a recommendation (e.g. due to ethical relevance or lack of research-possibilities). Expert opinions and consensus-based decisions were only allowed when the resolutions were highly relevant for clinical routine and there was lack of available evidence.

The guideline was reviewed and approved by 17 scientific societies. Reviewers were all independent peers.

All procedures are in adherence to the actualization protocol. The next regular update of the guideline is scheduled for 2018, although earlier modifications are permitted should significant new evidence arise.

## Management of delirium, analgesia, and sedation in adult intensive care

### Risk and prevention of ICU related delirium

Pain, stress, anxiety, and a disrupted sleep-wake cycle are common symptoms that occur in critically ill patients that increase the risk for ICU related delirium [3]. Delirium is one of the most common manifestations of cerebral dysfunction in critically ill patients, which affects not only short-term recovery (in terms of prolonged ventilation and length of stay, as well as increased mortality) [4], [5], [6], but also leads to cognitive long-term impairment, posttraumatic stress disorders (PTSD), and reduced quality of life [7]. These entities are part of the Post Intensive Care Unit Syndrome (PICS) that has been recently described in ICU survivors.

Aside from managing the risk factors, several effective pharmacological and non-pharmacological prevention strategies can be used to prevent or treat ICU delirium. In addition to baseline factors, the risk for delirium also comprises treatment associated factors, as well as psychological, social, and iatrogenic aspects [8].

The presence or development of risk factors for delirium shall be closely evaluated in order to ensure the prompt introduction of preventive measures. The non-pharmacological measures were shown to be particularly effective in the prevention of delirium, and shall therefore be implemented for all critically ill patients.

The excessive use of sedation shall be avoided, since a deep sedation were shown to deteriorate the clinical outcome [9]. A pharmacological prevention is to be reserved only for patients at high risk for delirium, and is not generally recommended (Table 1 (Tab. 1), Table 2 (Tab. 2), Table 3 (Tab. 3)).

### Monitoring of analgesia, sedation, delirium, anxiety, stress, and sleep

A basic concept of patient-oriented therapy in intensive-care is the definition of a patient-specific treatment goal, and the frequent assessment of the current status in order to promptly introduce or adjust interventions (Goal Directed Therapy). The definition and monitoring of treatment goals must be symptom-oriented, using validated scores and instruments. The use of such validated methods has a major impact on treatment: the systematic evaluation of pain, sedation, and delirium can significantly improve treatment of pain, reduce nosocomial infections, decrease duration of mechanical ventilation and hospitalization, as well as reduce mortality [10], [11].

**Figure 1:**
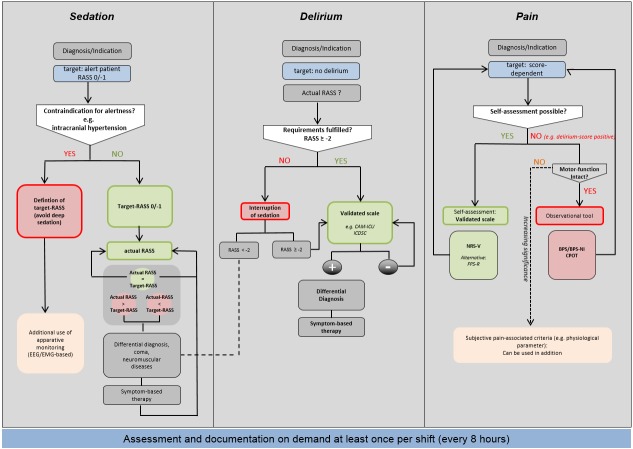
Algorithm for monitoring of sedation, delirium, and pain in adult patients RASS: Richmond Agitation, Sedation Scale; CAM-ICU: Confusion Assessment Method for the Intensive Care Unit; ICDSC: Intensive Care Delirium Screening Checklist; BPS: Behavioral Pain Scale; BPS-NI: Behavioral Pain Scale, not intubated; CPOT: Critical Care Pain Observation Tool; FPS-R: Faces Pain Scale, revised

Therefore, it should be a standard in all ICUs to define goals, measure, and document the current state of analgesia, sedation, and delirium once per shift (usually every 8 hours) with a validated clinical scores and instruments (Figure 1 (Fig. 1)).

The evaluation of anxiety with a validated scale is desirable, since anxiety is often not properly estimated, and thus not properly treated. There are short versions of standard psychological measurement instruments available (State-Trait Anxiety Inventory, state subscale [STAI-s], Brief Symptom Inventory Anxiety, subscale [BSI-A]) that allow a valid and reliable assessment of anxiety. From pain assessment, we know that one-dimensional self-assessment scales have proven to be especially practical to measure therapeutic needs and therapeutic response. From pain assessment studies, it is clear that the use one-dimensional self-assessment scales are particularly useful in evaluating the therapeutic needs and therapeutic response. Studies on anxiety show that similar scales (for example linear Visual Analogue Scale and the Faces Anxiety Scale) are also suitable to measure anxiety with good diagnostic validity [12]. Further studies are needed to clarify whether the controllability of anxiolysis can also be monitored with these scales.

Stress and disturbances of the sleep-wake cycle are further syndromes in ICU-patients that not only negatively affect recovery, but also constitute risk factors for serious complications. Nevertheless, there are currently no validated routine instrument for measuring stress and sleep, and the sole subjective assessment of vital signs by the ICU personnel is not suitable to monitor stress in critically ill patients [13]. Regarding the sleep-wake cycle of ICU patients, not only is there a lack of suitable monitoring procedures that can be routinely used, but also no valid evaluation of sleep stages in critically ill subjects [14] (Table 4 (Tab. 4), Table 5 (Tab. 5), Table 6 (Tab. 6), Table 7 (Tab. 7), Table 8 (Tab. 8), Table 9 (Tab. 9)).

### Treatment concepts

**Figure 2:**
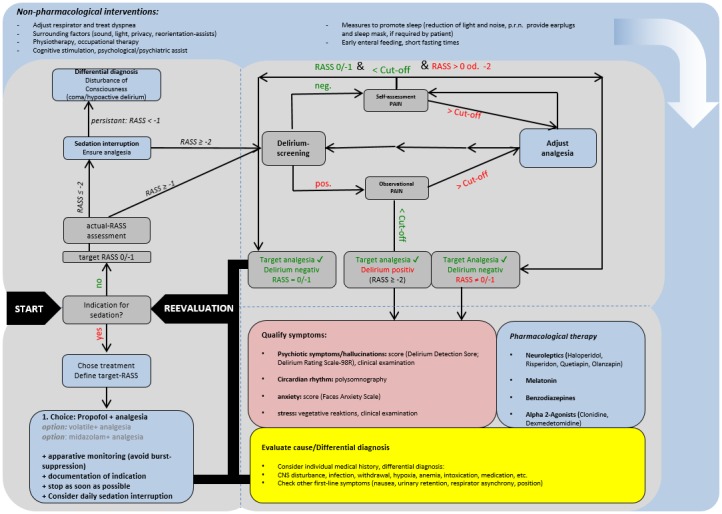
Symptom-based therapy and reduction of delirium, anxiety, stress, and protocol-based analgesia, sedation, and sleep-management in intensive care medicine Algorithm that focuses on a possible holistic management for adult critically ill patients. RASS: Richmond Agitation-Sedation Scale. Other scores (delirium, analgesia: observational/self-assessment), please, s.f. addendum at http://www.awmf.org/leitlinien/detail/ll/001-012.html.

See Figure 2 (Fig. 2).

#### Non-pharmacological concepts

See Table 10 (Tab. 10).

#### Analgesia

ICU-patients require an individualized pain management. Pain exerts considerable negative effects on recovery and is one of the most commonly reported stressors for ICU-patients. Therefore, a sufficient analgesia in all ICU patients – regardless of indication of sedation – should be ensured, and potentially painful procedures should be met with a preventive analgesic approach. In the ICU, the analgesia regimen is usually opioid based [15], [16], [17], [18], as the risk-benefit profile of non-opioid analgesics is still a matter of scientific discussion (low analgesic potential, but considerable side effects).

A combination with regional analgesia can be used [19], and a patient-controlled analgesia is recommended as soon as the patient is sufficiently awake (RASS 0/-1 and no delirium) [20] (Table 11 (Tab. 11)).

#### Sedation

The current evidence reveals that a measurable sedation should always be avoided, as long as there is no mandatory clinical indication for sedation [21], [22]. A deep sedation, also within the first 48 hours, is associated with increased mortality, prolonged mechanical ventilation, prolonged ICU-LOS, and hospitalization [23], [24], [25], [26]. Aside from specific indications (e.g. surgical indications, signs of increased intracranial pressure with impending herniation, or reduction of oxygen consumption in case of imminent hypoxia), the treatment goal should be an alert, cooperative patient who can tolerate the required interventions (RASS 0/–1). It is fundamental to achieve an adequate analgesia, which is accompanied by a specific treatment of possible symptoms, such as hallucinations, stress, and anxiety [27], [28], [29]. A recent systematic review revealed a decreased importance of daily sedation interruptions (DSIs), which is no longer considered superior to protocol-driven management of sedation [30]. The goal for light sedation should be set as early as possible, as the first 48 hours of sedation predict long-term outcome. Sedation should follow the principles of an “early goal directed therapy” with target-RASS 0/–1 (Table 12 (Tab. 12)).

#### Moderate or deep sedation

If the indication for a deep sedation is provided, the target RASS and the time of the next re-evaluation must be defined. Sedation should be performed with a combination of hypnotic and analgesic agents, whereas the choice of hypnotic agent should be appropriate to level of sedation and controllability desired. Aside from propofol and benzodiazepines, volatile anesthetics are also feasible options. In the absence of contraindications, DSI protocol (e.g., Spontaneous Awakening Trial, SAT) and Spontaneous Breathing Trials (SBT) should be carried out daily in patients with RASS ≤–2 [31] (Table 13 (Tab. 13)).

#### Symptom oriented sedative therapy

Aside from pain, the most common symptoms of critically ill patients are stress, anxiety, agitation, psychotic symptoms, and sleep disturbances, all of which require a targeted, symptom-oriented therapy. In addition to non-pharmacological strategies and a causal treatment, a pharmacological therapy may be necessary to control the symptoms. Alpha-2-agonists are available for stress-reduction and treatment of vegetative symptoms, and benzodiazepines can be used for anxiolysis (Table 14 (Tab. 14)).

#### Pharmacological therapy of delirium

Preventive measures for delirium are both safe and effective [32]. If delirium does occur, it should be treated promptly and symptomatically. The treatment of psychotic symptoms (with or without delirium) may include low-dose neuroleptics [33]. In addition, the use of alpha-2 agonists is also suitable for a symptom-orientated therapy of delirium [34], [35]. A special situation is the alcohol withdrawal delirium in the ICU, for which long-acting benzodiazepines should be considered [36] (Table 15 (Tab. 15)).

#### Weaning from mechanical ventilation

The new German S2k-Guidelines on “Prolonged Weaning” AWMF (020/015) provides recommendations regarding weaning from mechanical ventilation [37]. The management of analgesia, sedation, and delirium influence the weaning process significantly [38]. In order to start weaning as early as possible, it is useful to combine sedation and weaning protocols (Table 16 (Tab. 16)).

#### Treatment with neuromuscular blocking agents (NMBA)

There is no indication for a general use of a neuromuscular blockade during intensive care treatment. During neuromuscular blockade, patients cannot be awake, actively participate in their recovery, nor benefit from physiotherapy. When there is a specific indication for NMBAs, adequate analgesia and sedation should be provided [39]. Furthermore, the depth of blockade should be monitored [40], and the duration should be kept as short as possible (Table 17 (Tab. 17)).

#### Intra- and inter- hospital transports

Especially during intra- and inter-hospital transports, movements or changes in the position of the patient are potentially painful events. Therefore, a symptomatic therapy should be continued and possibly adjusted during a transport [41]. An inadequate sedation (over- and under-sedation) should be avoided at all times, as this can lead to an acute deterioration of neurological and cardiovascular parameters (Table 18 (Tab. 18)).

## Analgesia, sedation, and delirium management in special patients

### Pregnant and breastfeeding women in the ICU

The pharmacotherapy of pregnant and breastfeeding patients in the ICU is particularly challenging, as it must account for the effects of the drugs on the unborn or breast-fed child. Due to the lack of randomized-controlled trials, there is little evidence for pharmacotherapy of pregnant or breastfeeding women. Should there be an indication for opioids or sedatives during these periods, an adequate monitoring of the child is obligatory (Table 19 (Tab. 19)).

### End-of-life care

Impending death is a period that can be severely influenced by anxiety, stress, and pain. Dying patients require the same patient-centred care as other ICU patients. Frequent monitoring and sufficient pharmacotherapy should ensure the patient is free of discomfort, even if this leads to a acceleration of the dying process [42]. This guideline should be used and evaluated in the light and in-line with the “guideline of limitations to intensive care treatment” [43] and the “basic principles for medical palliative care”, published by the German Medical Association [44] (Table 20 (Tab. 20)).

### Patients with severe burn injury

Severe burn injuries lead to strong pain, possible hyperalgesia, as well as a prolonged hospitalization. Aside from an adequate basic level of analgesia, additional analgesics (local and systemic) and/or procedural sedation may be necessary when performing various procedures (e.g. dressing changes). There are multimodal concepts for the use of analgesics, adjuvants, and non-pharmacological strategies regarding pain management in patients with severe burns [45]. Especially for analgesia and sedation of burn-injured children, the use of standardized protocols and training programs should be used [46] (Table 21 (Tab. 21), Table 22 (Tab. 22)).

### Multiple trauma patients

The German AWMF-guideline registered under 012/019 provides recommendations regarding patients with multiple trauma [47]. There is no evidence for a clear superiority of a particular drug for analgesia in severely injured patients. Opioids (morphine, fentanyl, sufentanil, alfentanil, remifentanil), ketamine [48] (s-enantiomer, racemate), and adjuvant alpha-2-agonists (clonidine) are used routinely in the clinic. Due to an increased risk for adrenal insufficiency and numerous alternatives, etomidate should no longer be used for procedural sedation, and is no longer recommended in trauma patients. Nevertheless, there is no evidence that the administration of etomidate has long-term effects on outcome [47], [49] (Table 23 (Tab. 23)).

### Patients with intracranial hypertension

A main focus of the intensive care management of patients with severe traumatic brain injury is the reduction of secondary damage. Although there is little evidence that sedation directly lowers intra-cranial-pressure (ICP), the acute treatment of patients with elevated ICP starts with a deep sedation (RASS-5). There are no validated monitoring systems for this patient subgroup, so the general scores and monitoring instruments are used [50]. A frequent neurological examination is obligatory. The choice of sedatives should be in-line with recommendations made in the general part. An ideal analgesic/sedative for patients with elevated intracranial pressure should decrease ICP while sustaining an adequate cerebral perfusion pressure, as well as maintain cerebral hemodynamics, including cerebral autoregulation. Additionally, it should reduce the cerebral metabolic rate for oxygen (CMRO2), have anticonvulsive and neuroprotective properties, and should allow short wake-up times for the assessment of patients after a brief infusion interruption [51] (Table 24 (Tab. 24)).

### Cardiac surgery

Fast-track concepts include a 2-hour sedation phase after uncomplicated cardiac surgery. In this setting, numerous analgesia and sedation protocols have proven to be advantageous [52], and fast-track strategies seem to reduce the incidence of postoperative delirium [53].

Delirium in cardiac surgical patients is associated with a higher mortality [54], so that a delirium screening with a validated tool – as generally recommended – is especially important [55], [56] (Table 25 (Tab. 25)).

### Patients on extracorporeal life support systems (ECLS)

There is a grey area regarding the level of sedation for patients on ECLS, where safety-aspects and the ability to positively influence recovery must be balanced. Patients on ECLS have numerous delirium risk-factors. Hyperactive delirium or agitation can be life-threatening for these patients, so that a consequent monitoring and a symptomatic therapy of stress, anxiety, delirium, pain, and insomnia is essential to safely achieve a target RASS of 0 [57], [58], [59], [60], [61] (Table 26 (Tab. 26)).

### Special positioning of patients

Positioning therapy is used for prophylaxis and treatment of respiratory dysfunctions [62], and requires an individual sedation target. Changes of the position frequently represent a challenge for the symptomatic treatment of anxiety, stress, and pain. Therefore, a symptom-orientated therapy should be adapted for changing demands during positioning therapy. Though a deep sedation may be indicated for patient repositioning, [63], [64], an excessive sedation should be avoided.

## Analgesia, sedation, and delirium management in children

### Monitoring

For the monitoring of analgesia, sedation, and delirium in children, there are validated monitoring scales are that take developmental stage into account [65]. Also in the pediatric intensive care, adequate monitoring and individual therapy goals are essential for successful patient-oriented care (Table 27 (Tab. 27), Table 28 (Tab. 28), Table 29 (Tab. 29)).

Generally, children aged ≥3 years are able to evaluate pain-levels. Even in children, a self-assessment of pain is superior to observational scales [66], and the *Faces Pain Scale-revised* has been well-established as a valid monitoring tool [67]. If children are unable to assess their pain, there are several age-appropriate observational pain assessment scales. However, both in very premature infants as well as in children and adolescents with neurocognitive impairment, those instruments have a limited value and tend to systematically underestimate pain [68], [69], [70].

Combined pain and distress sedation scales have been validated for the monitoring of sedation in children. For premature and full-term neonates, the *Neonatal Pain, Agitation and Sedation Scale* (N-PASS) is available. For infants and toddlers, the *COMFORTneo Scale* and the *Comfort-B Scale* are available. Additionally, there are special scales for assessing opioid or sedative withdrawal following a continuous therapy.

Children can also suffer from delirium, and their symptoms are often misinterpreted. The pediatric critical care community has a need for a systematic delirium screening with validated tools [71], [72], [73], [74].

### Treatment strategies in children

Critically ill children – like adults – require an individual pain therapy adapted to their current situation. This includes multimodal therapy strategies for opioids, non-opioids, and regional analgesia, as well as for local anesthetics, co-analgesics and non-pharmacological procedures (Table 30 (Tab. 30), Table 31 (Tab. 31), Table 32 (Tab. 32), Table 33 (Tab. 33)). When regarding analgesia in children, it is important to consider that pharmacokinetics and pharmacodynamics differ with age. Additional and supportive procedures for analgesia are also recommended for children. There are different non-pharmacological procedures available that can be used for co-analgesia, per example the administration of oral glucose, non-nutritive suction for neonates, or virtual reality for pediatric burn patients.

Children require sedation, sometimes continuously, in order to undergo certain diagnostic and therapeutic procedures. For sedation in children, special personnel and structural prerequisites are required. Oversedation should be avoided as always, and careful titration is required to keep dosages as low as possible.

There is still a high demand for research regarding pediatric delirium. In principle, it is essential to detect delirious symptoms as early as possible and identify and neutralize potential causes. Current evidence revealed a combination of psychological, social (presence of family, toys, pictures of home, normal day-night rhythm, etc.), and pharmacological interventions to be effective [75].

## Analgesia, sedation, and delirium management in elderly patients

The “clinical age” is determined by the biological age, frailty, comorbidities, long-term medication, and external influences. The ageing of the cardiovascular, respiratory, renal, and nervous-systems lead to changes in pharmacodynamics and kinetics. The inherent age cannot be based on a chronological age alone (Table 34 (Tab. 34), Table 35 (Tab. 35)).

Elderly patients lack the resources to compensate for delirium-associated complications, thus a frequent and active screening for delirium is paramount. In principle, all monitoring instruments used for the adult patients may be used for elderly patients. To evaluate pain in patients with cognitive impairment or dementia, tools such as Faces Pain Scale, PAINAD-scale as well as the BESD-scale (German scale) are available.

Preventive measures such as reorientation, visual and hearing aids are especially indicated for elderly patients [76]. Regarding the treatment of delirium, a symptomatic treatment should consider the delirogenic effects of long-acting benzodiazepines [77], the cardiac side effects of neuroleptics, and the use of appropriately cautious dosages [78], [79], [80]. For the treatment of delirium, melatonin or melatonin-analogues should be considered at night to reduce the incidence and duration of delirium [81].

## Economy, quality assurance and implementation of the guideline

In terms of quality assurance, management of analgesia, sedation and delirium in the ICU should be conducted according to guidelines and subject to a continuous quality verification [82].

This includes the regular training of personnel in the implementation of the guidelines [83]. Special consideration for regional characteristics and internal Standard Operating Procedures improved the integration of guideline recommendations [83]. As a follow-up to the surveys of 2002 and 2006, a current survey on the current implementation of the S3-guideline in clinical routine is being carried out and will be published. Before the next guideline update process, an additional survey will assess the level of implementation (Table 36 (Tab. 36)).

## Notes

### Extended version of the guideline

The German extended version, patient version, and methodology report are available under http://www.awmf.org/leitlinien/detail/ll/001-012.html.

### Authorship

DAS Taskforce 2015: The task-force members (authors) are listed in alphabetical order.

Chairing medical societies: German Society of Anaesthesiology and Intensive Care Medicine (DGAI) and German Interdisciplinary Association for Intensive Care and Emergency Medicine (DIVI) with 15 participating medical societies^$^. All societies or their executive boards consented on the final version of the guidelines.

1. German Society of Anaesthesiology and Intensive Care Medicine (DGAI)
2. German Interdisciplinary Association for Intensive Care and Emergency Medicine (DIVI)
3. German Society of Surgery (DGCH)^$^
4. German Society for Specialised Nursing and Allied Health Professions (DGF)^$^
5. German Society of Geriatrics (DGG)^$^
6. German Society for Gynecology & Obstetrics (DGGG)^$^
7. German Society of Haematology and Oncology (DGHO)^$^
8. German Society of Midwifery Science (DGHWi)^$^
9. German Society of Internal Medicine Intensive Care (DGIIN)^$^
10. German Society of Neurosurgery (DGNC)^$^
11. German Society of Neurology (DGN)^$^
12. German Association for Psychiatry, Psychotherapy and Psychosomatics (DGPPN)^$^
13. German Sleep Society (DGSM)^$^
14. German Society for Thoracic and Cardiovascular Surgery (DGTHG)^$^
15. German Pain Society (DGSS)$
16. German Association for Physiotherapy (ZVK)^$^
17. German Society of Neonatology and Pediatric Intensive Care (GNPI)^$^

### Conflicts of interest

The declarations of conflict of interest from all participants can be viewed upon request and are published on the AWMF homepage. 

### Funding

This guideline has been funded independently of interest groups by the DGAI. 

### Acknowledgements

We thank all participating societies for their work, their outstanding commitment and the sound review of the guideline before publication. Our special thanks to Rudolf Mörgeli for the thorough correction of the English version of the guideline.

## Erratum

In the initial publication the author Irene Harth was erroneously omitted in the html version.

## Figures and Tables

**Table 1:**
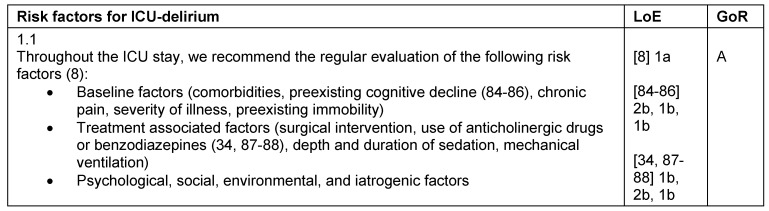
Risk factors for ICU-delirium

**Table 2:**
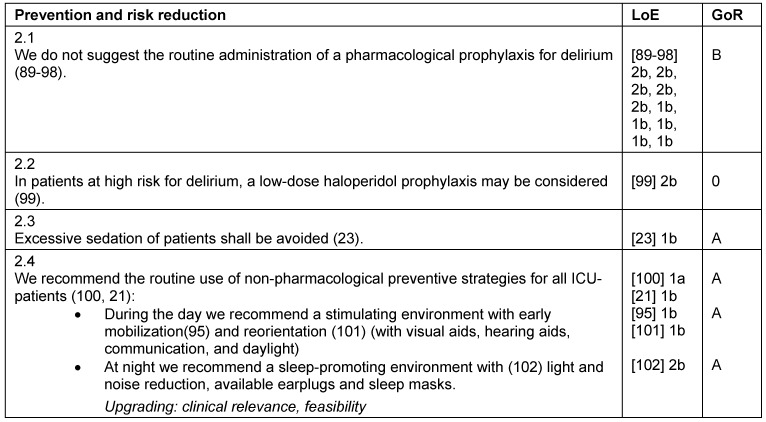
Prevention and risk reduction

**Table 3:**
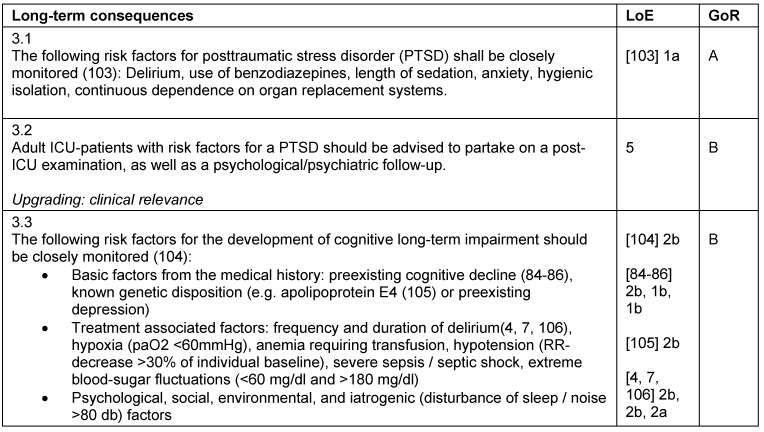
Long-term consequences

**Table 4:**
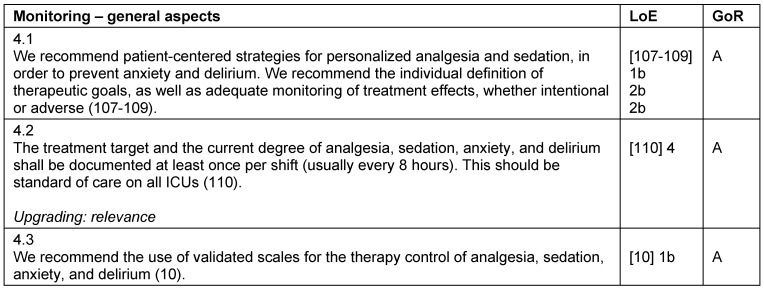
Monitoring – general aspects

**Table 5:**
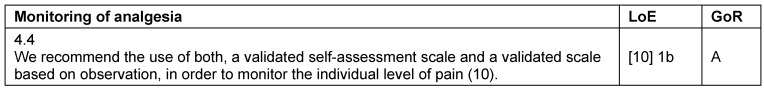
Monitoring of analgesia

**Table 6:**
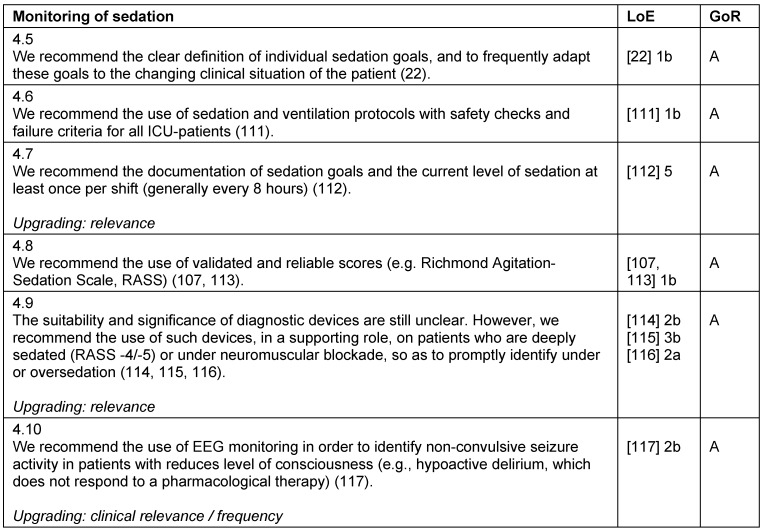
Monitoring of sedation

**Table 7:**
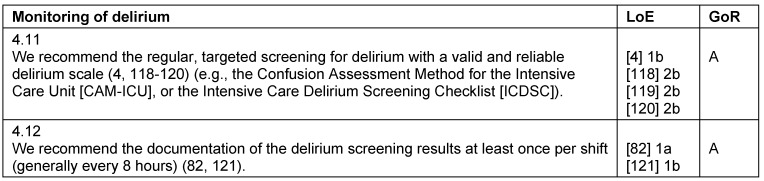
Monitoring of delirium

**Table 8:**
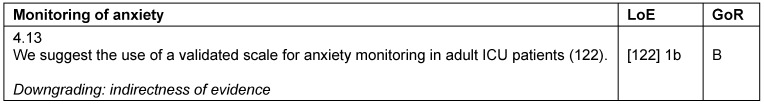
Monitoring of anxiety

**Table 9:**
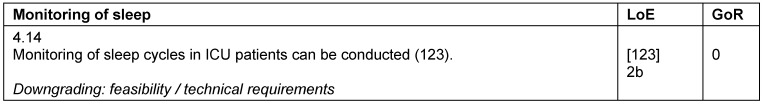
Monitoring of sleep

**Table 10:**
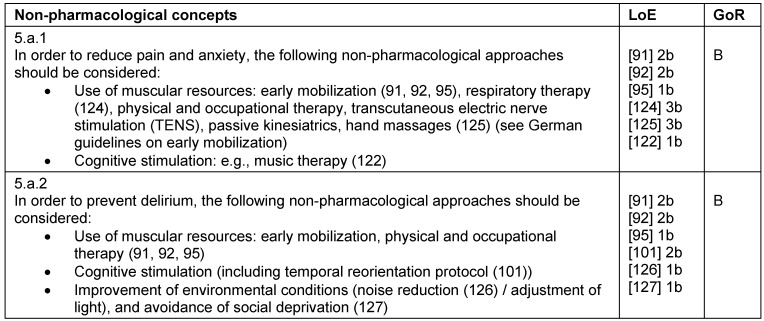
Treatment concepts – non-pharmacological concepts

**Table 11:**
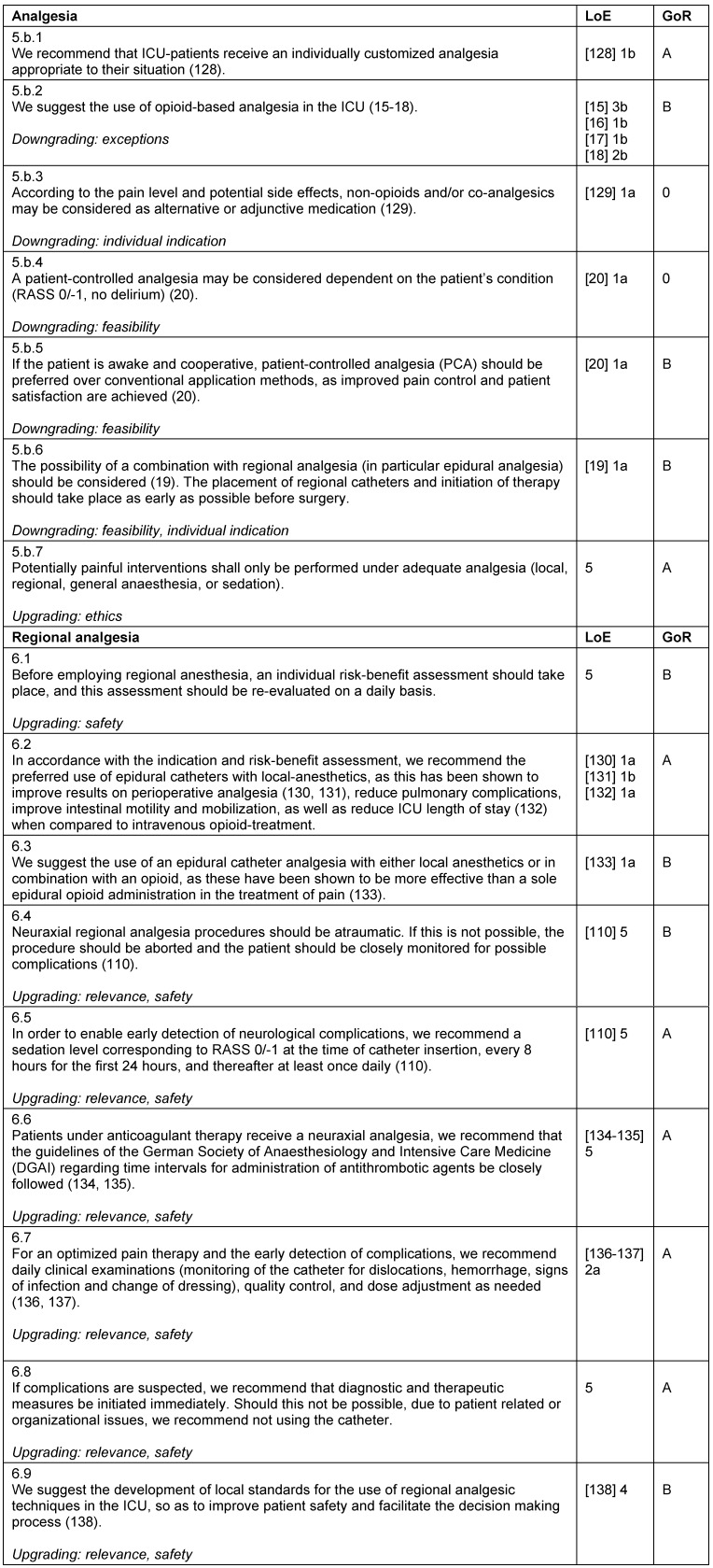
Analgesia and regional analgesia

**Table 12:**
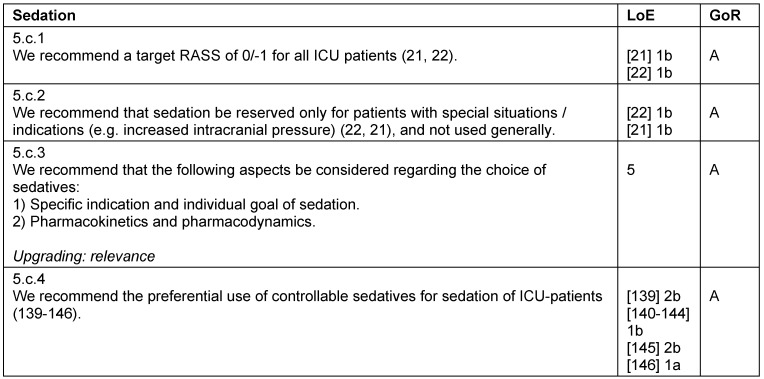
Sedation

**Table 13:**
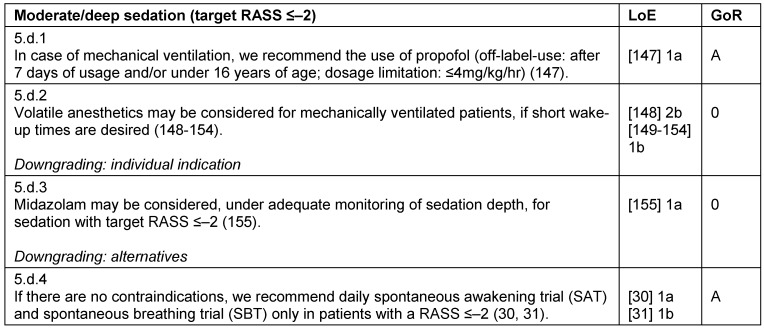
Moderate/deep sedation

**Table 14:**
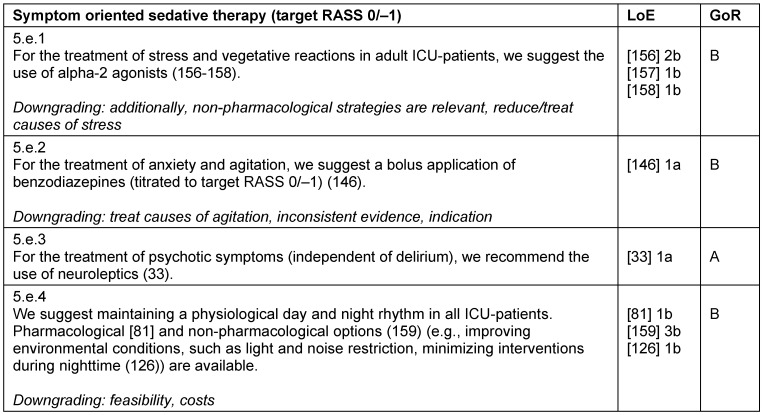
Symptom oriented sedative therapy (target RASS 0/–1)

**Table 15:**
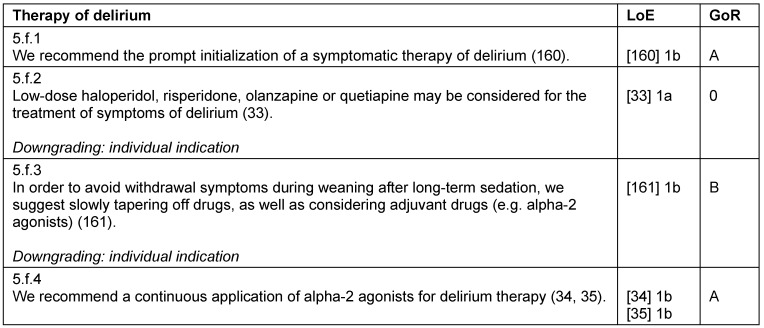
Therapy of delirium

**Table 16:**
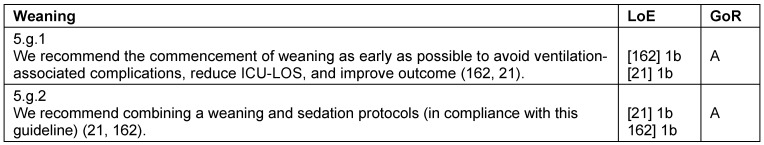
Weaning from mechanical ventilation

**Table 17:**
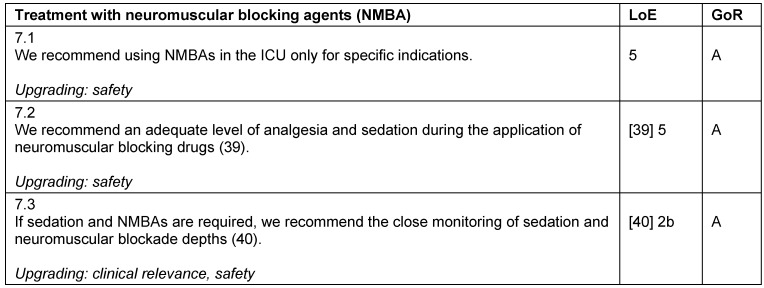
Treatment with neuromuscular blocking agents (NMBA)

**Table 18:**
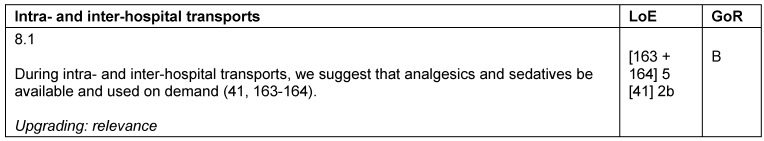
Intra- and inter-hospital transports

**Table 19:**
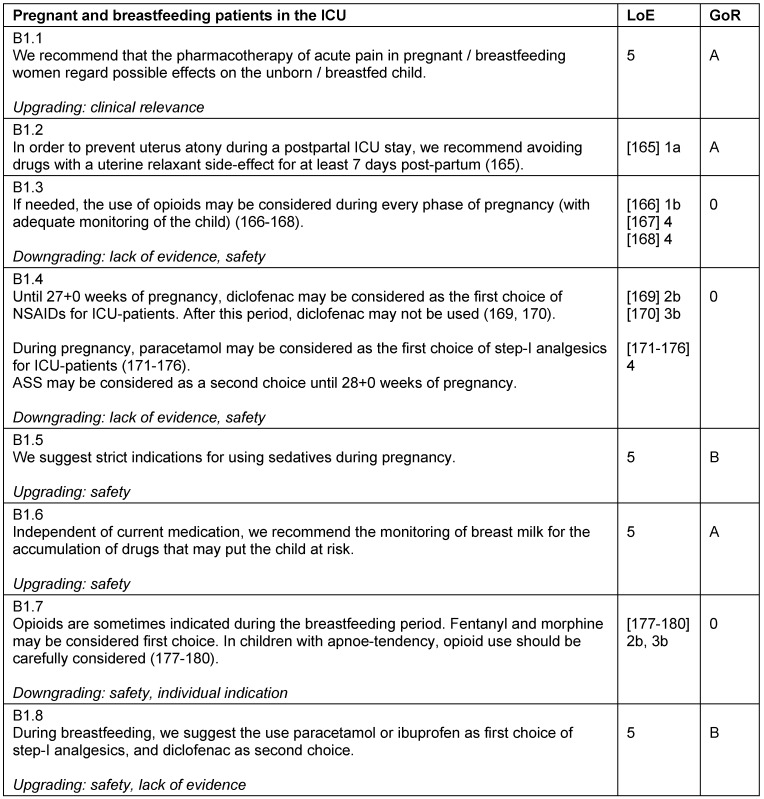
Pregnant and breastfeeding patients in the ICU

**Table 20:**
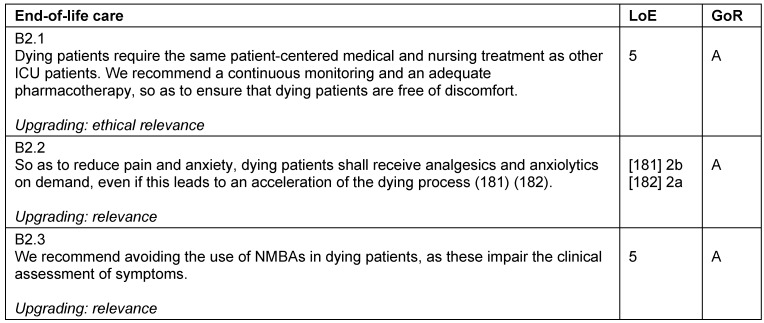
End-of-life care

**Table 21:**
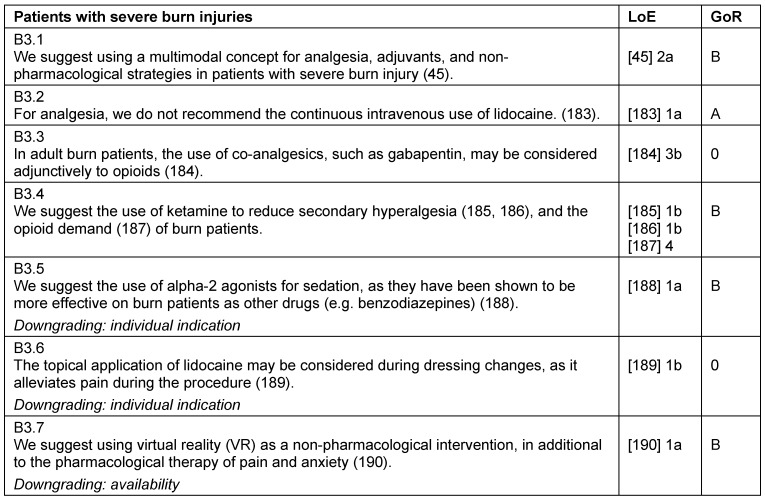
Patients with severe burn injuries

**Table 22:**
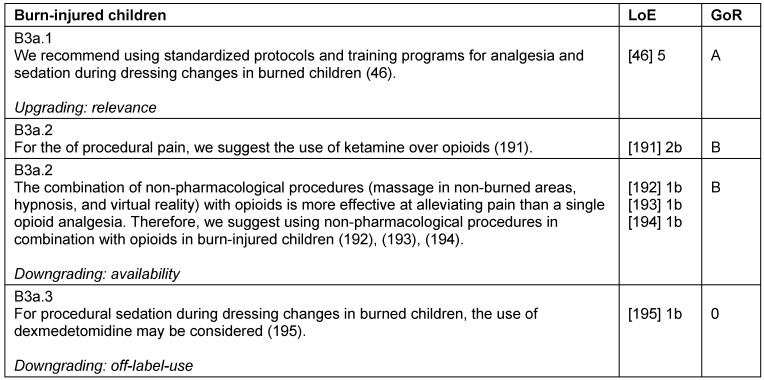
Burn-injured children

**Table 23:**
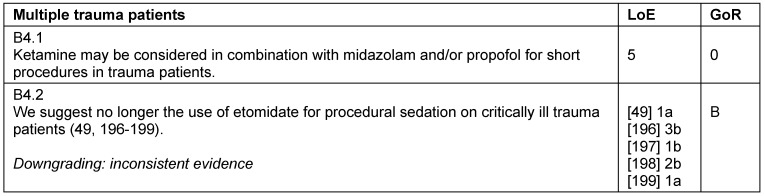
Multiple trauma patients

**Table 24:**
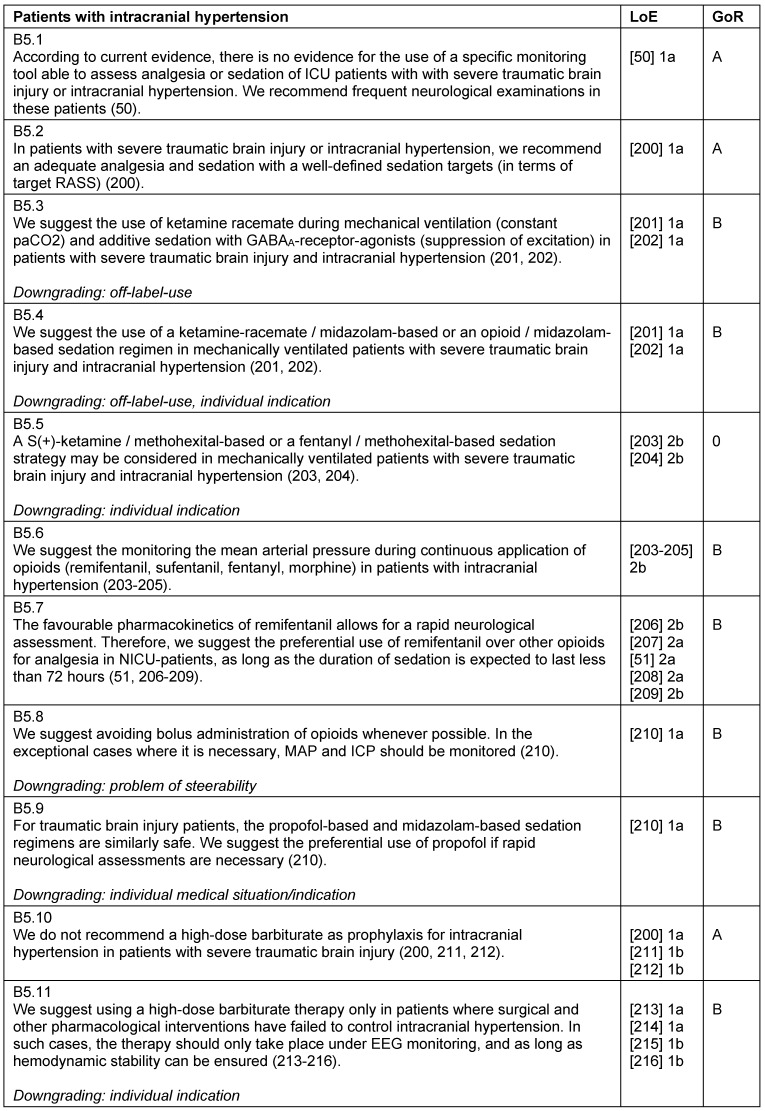
Patients with intracranial hypertension

**Table 25:**
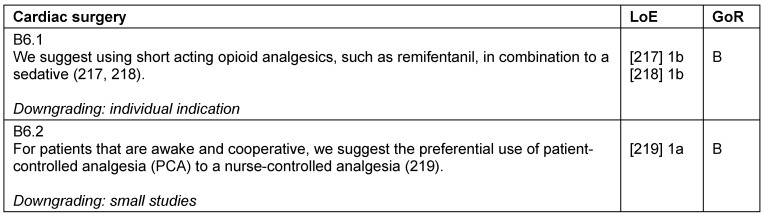
Cardiac surgery

**Table 26:**
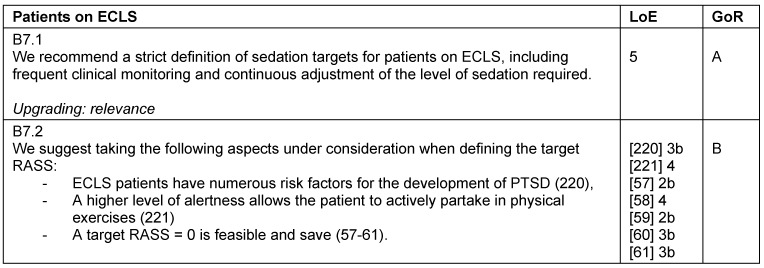
Patients on extracorporeal life support systems (ECLS)

**Table 27:**
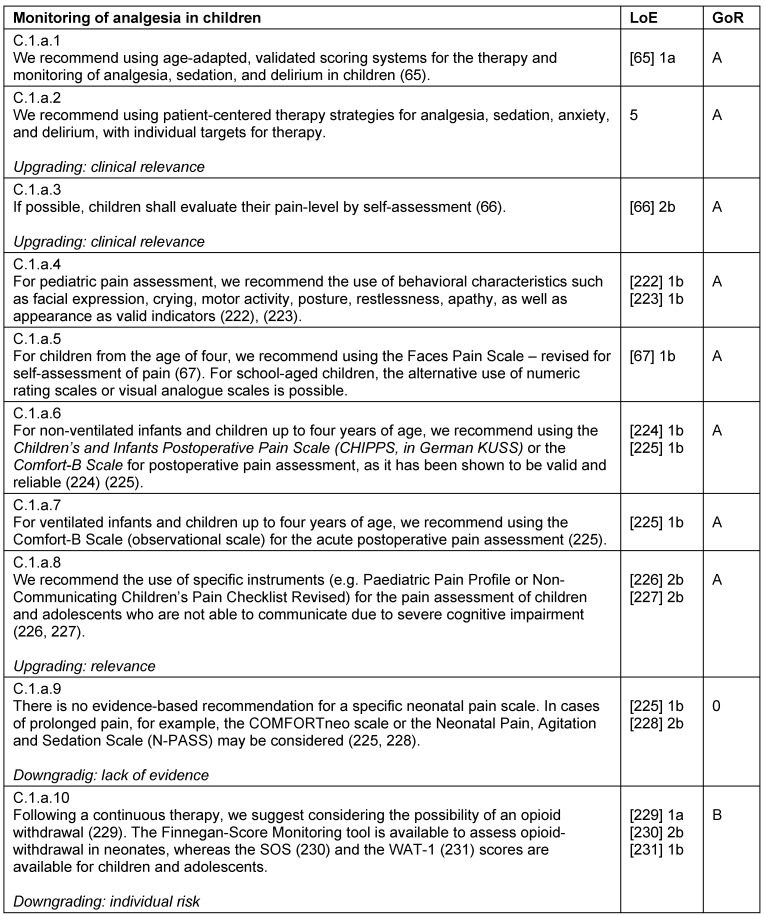
Monitoring of analgesia in children

**Table 28:**
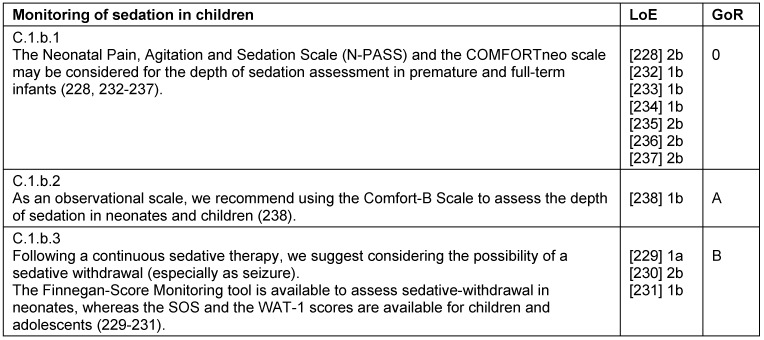
Monitoring of sedation in children

**Table 29:**
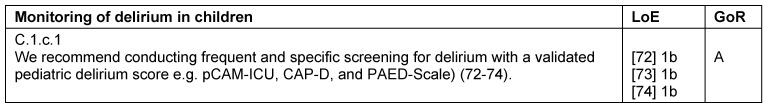
Monitoring of delirium in children

**Table 30:**
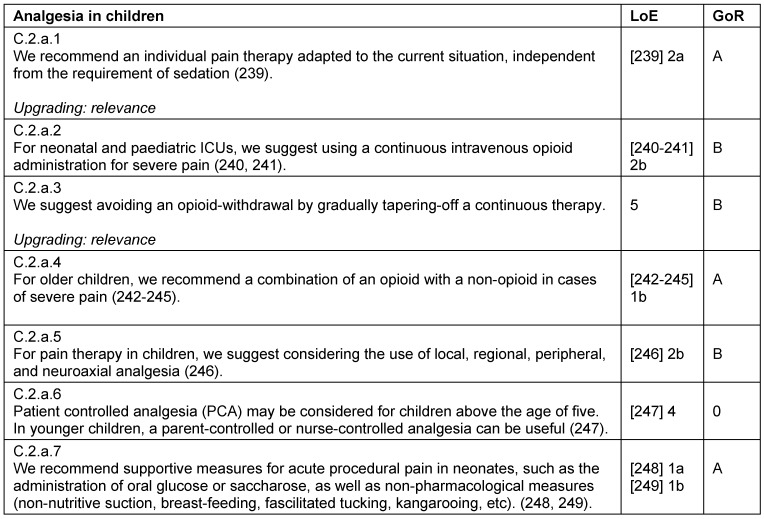
Analgesia in children

**Table 31:**
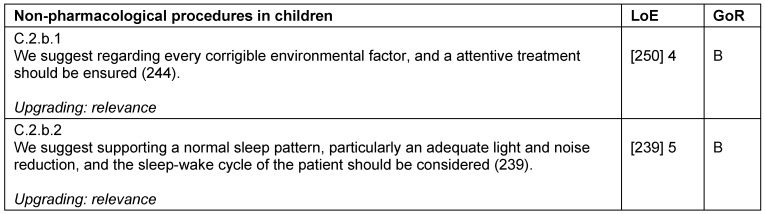
Non-pharmacological procedures in children

**Table 32:**
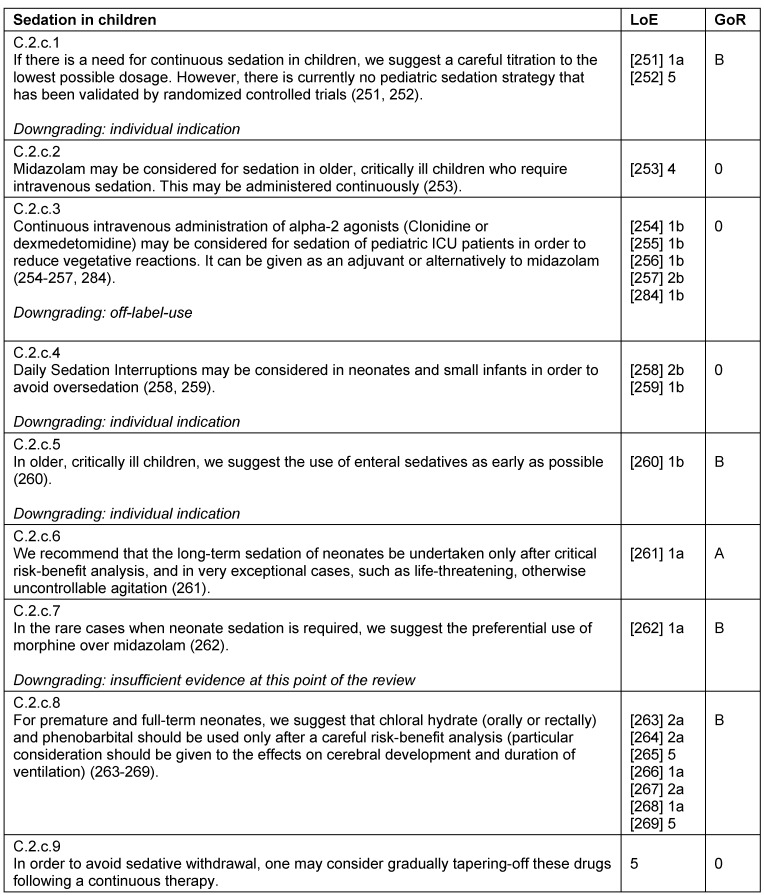
Sedation in children

**Table 33:**
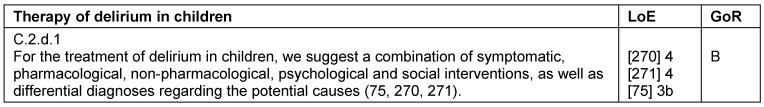
Therapy of delirium in children

**Table 34:**
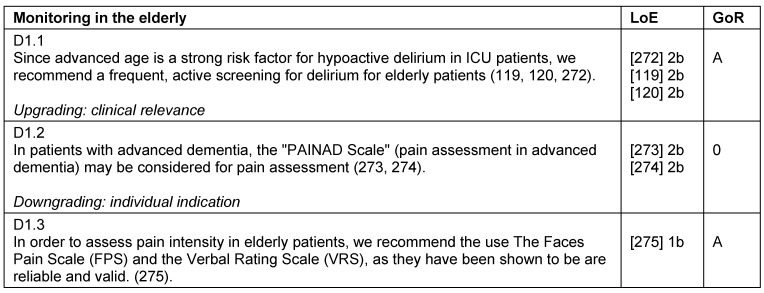
Monitoring in the elderly

**Table 35:**
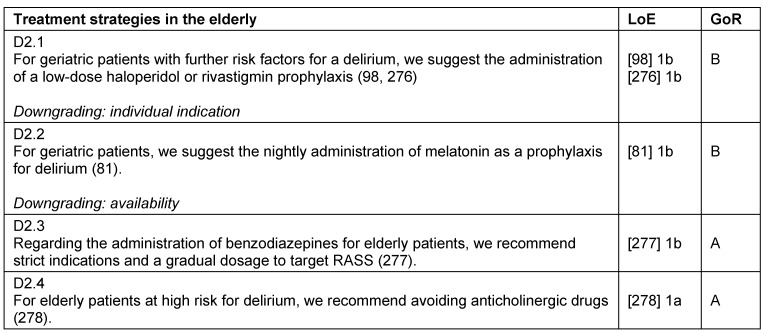
Treatment strategies in the elderly

**Table 36:**
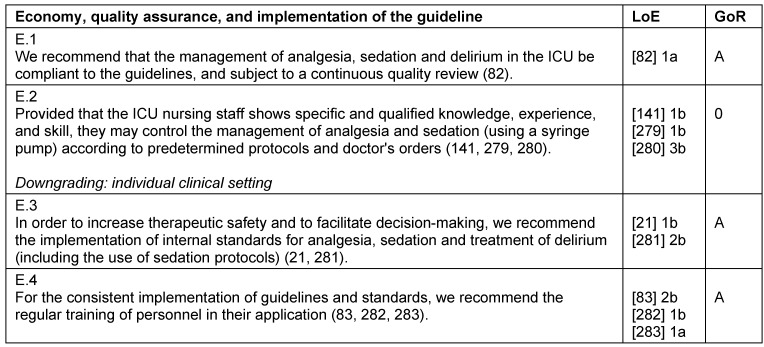
Economy, quality assurance, and implementation of the guideline
